# Editorial: Artificial intelligence and bioinformatics applications for omics and multi-omics studies

**DOI:** 10.3389/fgene.2024.1371473

**Published:** 2024-01-30

**Authors:** Angelo Facchiano, Dominik Heider, Margherita Mutarelli

**Affiliations:** ^1^ Institute of Food Sciences, National Research Council (CNR), Avellino, Italy; ^2^ Department of Mathematics & Computer Science, University of Marburg, Marburg, Germany; ^3^ Institute of Applied Sciences and Intelligent Systems, National Research Council (CNR), Pozzuoli, Italy

**Keywords:** artificial intelligence, multi-omics, systems biology, biomedical data science, machine learning

## Introduction

The omics sciences have revolutionized research in areas such as biology, biotechnology, medicine, and agri-food sciences. The production of large-scale datasets has led to strong demand for appropriate computational tools for their management, analysis, and interpretation. In the era of Big Data, this need has surged. The use of artificial intelligence is now widespread in the biomedical field. Of enormous impact are recent developments in the field of protein three-dimensional structure prediction, once considered achievable only with experimental techniques, however showing many limitations. The application prospects in all domains of molecular biology, genomics, and omics sciences are now a tangible reality.

Following up on a Research Topic already carried out in past years (Chicco et al., 2020), we introduce a new Research Topic of articles to present Artificial Intelligence and new bioinformatics applications and computational approaches for analyzing omics data, or the application of existing tools, toward a more complete interpretation of biological phenomena, with applications in personalized medicine and biotechnology.

The Research Topic includes 12 articles, of which 9 are classified as Original Research, 2 as Brief Research Report, and 1 as Methods.

## Original research articles

Sun et al. introduced a new machine learning model, which integrates multi-omics data for accurate cancer subtype recognition. This model combines an adversarial generation network and the self-attention mechanism. By learning from multi-omics data, the new model efficiently identifies cancer subtypes, outperforming traditional methods. The study demonstrates its effectiveness across various cancer datasets, highlighting its potential for improving cancer diagnosis and treatment strategies.

Ochoa and Hernandez-Lemus explored the functional impact of multi-omic interactions in breast cancer subtypes. They propose a comprehensive analysis framework to semi-automatically generate network models of regulatory constraints influencing biological functions. By analyzing multi-omics data, they identified significant functions enriched in various breast cancer molecular subtypes, highlighted new regulatory features, and demonstrated the capability of multi-omic regulatory networks to provide reliable models for understanding the connections between omics, thereby aiding in systematic generation of mechanistic hypotheses in cancer biology.

Liu et al. introduced a novel model for classifying gastric cancer subtypes. It utilizes a residual graph convolutional network, combining multi-omics data and patient similarity networks. The study demonstrates that the new approach significantly outperforms traditional methods in predictive performance. This approach offers potential advancements in understanding gastric cancer subtypes and could assist in developing more targeted treatments.

Luo et al. presented SupCAM, a method that improves the identification of chromosome clusters for karyotyping. The new approach involves pre-training the backbone network with supervised contrastive learning on ChrCluster, incorporating variable image composition by category, and introducing self-marginal loss. Fine-tuning the network results in a final model, with SupCAM achieving a 94.99% accuracy on the ChrCluster dataset, surpassing previous methods.

Zheng et al. proposed a machine learning method for predicting if pairs of enhancers and promoters physically interact. They built a model called HARD from the names of the four (epi)genomic signals included: the histone modification H3K27ac and ATAC-seq to represent chromatin accessibility, RAD21 subunit of cohesin that is important in loop formation and the Distance between the promoter and the enhancer and classify them using a random forest algorithm. The method was tested on enhancer-promoter interaction benchmarks from the BENGI database (Moore et al., 2020) and compared with two existing methods outperforming them in the majority of measures, thus proving to be a useful new approach to this important although complex task.

Ettetuani et al. presented article focused on gene expression analysis, using *p*-values to identify significant genes, gene ontology terms and similarity scores to understand biological pathways, regulation, and gene networks, and machine learning for gene prioritization. The study proposes using deep neural network algorithms for gene clustering based on regulatory pathways. The work validates findings through the detection of genetic interactions. Specific tissues with normalized gene expression and occurrence frequencies are considered, particularly in the context of glomerular diseases. The results highlight the relevance of genes like EGR1, IL33, BMP2, and SLAMF8 in glomerular diseases.

Wang et al. proposed a novel framework for predicting Alzheimer’s disease risk genes by considering spatial and temporal features of gene expression data. Utilizing gene expression data from various tissues and age groups, a support vector machine model is developed. The work identified 19 crucial features from an initial set of 64, and 15 potential risk genes with a probability exceeding 90%, offering a promising approach for understanding Alzheimer’s disease genetic etiology.

Jia et al. presented a deep learning tool to predict N4-acetylcytidine (ac4C), a post-transcriptional RNA modification highly conserved with a relevant function in transcription regulation and protein translation and associated with several human diseases. The authors tested different encoding approaches and classification models and found that a simple one-hot encoding and a downsampled ensemble deep learning network consisting of a modified DenseNet and Squeeze-and-Excitation Networks with a convolutional residual structure in parallel with the dense block gave the best performance results. The model outperformed two existing methods in a fair comparison, proving it is a promising new resource in predicting this important nucleoside modification.

The manuscript by Rahmani et al. described the development of an AI-based R package called MBMethPred to classify childhood medulloblastoma (MB) subtypes from DNA methylation and gene expression data. The two data types were combined using a similarity network fusion approach and feature selection was performed with random forests. The authors then applied six different machine-learning algorithms for subtype predictions, all scoring very good with a variety of performance measures and the selected biomarkers were challenged for biological and clinical relevance using survival and network analysis. The study represents a useful advancement towards the goal of accurate classification of molecular subgroups in MB patients that are vital to choose the best therapeutical plans for them.

## Methods articles

Klinkhammer et al. presented an article describing the development of a boosting algorithm, called snpboost, for creating polygenic risk scores (PRS) directly from genetic data, with the aim of improving predictive accuracy in clinical risk stratification. The approach efficiently addresses the high-dimensional nature of genotype data and outperforms other methods in terms of predictive performance and computational time.

Klau et al. focused on improving disease risk prediction using polygenic risk scores (PRS). They investigated whether incorporating multiple PRS from different diseases and applying machine learning models can enhance predictive accuracy compared to traditional single-PRS models using regression. Their results show that multi-PRS models, especially when combined with deep learning techniques, significantly outperform single-PRS models in predicting risks for diseases like cancer, diabetes, and cardiovascular diseases. This advancement could lead to more effective disease prediction and personalized medicine approaches.

## Brief research report article

Waechter et al. investigated the effectiveness of two different 16S rRNA primer sets for sequencing human fecal microbiomes using the Nanopore platform. They compared the conventional 27F primer included in the 16S Barcoding Kit by Oxford Nanopore Technologies and a more degenerate 27F primer. The study reveals significant differences in the detection of taxonomic diversity and relative abundance of various taxa between these primer sets. The more degenerate primer set appears to provide a more accurate and diverse representation of the fecal microbiome compared to the conventional primer set.
